# Synergistic Solubilization of Phenanthrene by Mixed Micelles Composed of Biosurfactants and a Conventional Non-Ionic Surfactant

**DOI:** 10.3390/molecules25184327

**Published:** 2020-09-21

**Authors:** Jianfei Liu, Yuru Wang, Huifang Li

**Affiliations:** School of Civil Engineer, Henan Polytechnic University, Jiaozuo 454003, China; wangyuru1996@126.com (Y.W.); lihuifang1009@163.com (H.L.)

**Keywords:** mixed surfactant, solubilization, phenanthrene, rhamnolipids

## Abstract

This study investigated the solubilization capabilities of rhamnolipids biosurfactant and synthetic surfactant mixtures for the application of a mixed surfactant in surfactant-enhanced remediation. The mass ratios between Triton X-100 and rhamnolipids were set at 1:0, 9:1, 3:1, 1:1, 1:3, and 0:1. The ideal critical micelle concentration values of the Triton X-100/rhamnolipids mixture system were higher than that of the theoretical predicted value suggesting the existence of interactions between the two surfactants. Solubilization capabilities were quantified in term of weight solubilization ratio and micellar-water partition coefficient. The highest value of the weight solubilization ratio was detected in the treatment where only Triton X-100 was used. This ratio decreased with the increase in the mass of rhamnolipids in the mixed surfactant systems. The parameters of the interaction between surfactants and the micellar mole fraction in the mixed system have been determined. The factors that influence phenanthrene solubilization, such as pH, ionic strength, and acetic acid concentration have been discussed in the paper. The aqueous solubility of phenanthrene increased linearly with the total surfactant concentration in all treatments. The mixed rhamnolipids and synthetic surfactants showed synergistic behavior and enhanced the solubilization capabilities of the mixture, which would extend the rhamnolipids application.

## 1. Introduction

Hydrophobic organic compounds are commonly found in soil and groundwater in industrialized areas. Among many hydrophobic organic compounds, polycyclic aromatic hydrocarbons have caused widespread concern on account of their poisonousness to humans and to ecosystems. Polycyclic aromatic hydrocarbons (PAHs) are a group of organic compounds composed of two or more aromatic rings fused with carbon and hydrogen atoms [1]. Due to their poor aqueous solubility, high hydrophobicity and strong sorption to soil particles or sediment matrix, PAHs are difficult to recover from the subsurface system and represent a long-term source of soil and aquifer contamination [2,3].

Based on this different principle, various remediation methods for PAH-contaminated sites have been proposed [4,5,6]. Surfactant-enhanced remediation has received considerably more attention among these approaches [7,8]. Surfactants consist of amphiphilic molecules and comprise both the hydrophilic head and hydrophobic tail simultaneously. When the surfactant concentration is above its critical micelle concentration (CMC), the micelles with hydrophilic surfaces and lipophilic core can dramatically improve the solubility of hydrophobic organic compounds in the water phase, thereby further promoting the desorption of contaminants from the soil. It should be noted that CMC is the parameter that refers to the pure water system and the actual CMC value in soil is much higher; thus, the surfactant concentration is much higher to improve the soil washing efficiency [9]. A large number of studies have been conducted on the use of surfactants for the enhancement of the solubilization capabilities of hydrophobic organic compounds, which are associated with the structures of the surfactant and the solute [10,11,12,13]. In general, nonionic surfactants have a greater solubilization potential than anionic surfactants, and the mixtures of anionic/nonionic surfactants have shown solubility even higher than that of each individual surfactant. However, there are concerns related to the fate of synthetic surfactants after soil remediation. Surfactants themselves also cause a number of environmental concerns due to their toxicity and long degradation times [14]. Therefore, in recent years, there has been a trend towards the replacement of synthetic surfactants by biosurfactants with lower toxicity and higher biodegradability. Produced by microorganisms, biosurfactants are surface-active amphiphilic biomolecules that have higher biodegradability, and are expected to be more environmentally compatible. Biosurfactants can reduce the toxicity of some hydrophobic organic compounds by a combination of toxin accumulation in biosurfactant micelles, which could entrap xenobiotics inside and results in xenobiotics being not accessible for microbes [15]. Specific interactions occurred between rhamnolipids and chlorinated phenols, which limit their toxicity and enhance biodegradation of diesel fuel hydrocarbons [16]. Despite the wide range of application of the class of biosurfactants, the greater difficulties of their large-scale production and purification have limited the spread of their use in practice [17]. Thus, it is necessary to study the properties and ecological effects of mixtures of biosurfactants with conventional surfactants. The research conducted on the potential for expanding the applications of biosurfactants by mixing them with synthetic surfactants is limited. For example, Chen et al. [18] used small-angle neutron scattering measurements to determine the surfactant structure of a mixture of rhamnolipids and sodium dodecyl 6-benzene sulfonate. Liley et al. [19] examined the self-assembly behavior and aggregation number of rhamnolipids mixed with different conventional surfactants. However, it should be pointed that biosurfactant also have negative effects on the biodegradation of hydrophobic substrates. Regardless of synthetic or natural surfactant, surfactants itself have some negative aspects for their physical and chemical properties. Łukasz et al. [20] found that the biodegradation of rhamnolipids is prior to that of that diesel oil. Furthermore, the addition of rhamnolipids to diesel oil-contaminated soil samples could enhance the phytotoxicity. Rhamnolipids might be consumed as preferential carbon source and rhamnolipids-mediated biodegradation is not a general method in soil remediation [21].

In this paper, the interfacial behavior and solubilization capabilities of a mixture of biosurfactants with a synthetic surfactant was studied. Biosurfactant is mainly produced by microorganisms and have various structures [22]. The most used biosurfactants are the rhamnolipids, which are engendered by the bacterium *Pseudomonas aeruginosa*. Rhamnolipids (Rham) are surface-active glycolipids of microbial origin [23]. The nonionic surfactant octylphenol polyethoxylene, Triton X-100 (TX100) in the experiments was selected due to its better removal efficiency in soil and underwater remediation and its mole weight, which is comparable to that of rhamnolipids [24]. As a typical polycyclic aromatic hydrocarbon, phenanthrene was selected in the study.

The goal of this study is to reduce the use of synthetic surfactant. The solubilization capabilities of rhamnolipids and synthetic surfactant mixtures for the application of surfactant-enhanced remediation was investigated. Further, the mechanism of possible synergism was analyzed based on the CMC values and solubility data obtained of the single and mixed surfactants. In addition, the influential factors, including pH, ionic strength, and acetic acid concentration were studied.

## 2. Materials and Methods

### 2.1. Materials

The biosurfactant rhamnolipids was purchased from Hunan Zhijin Biotech Co., Ltd., Changsha, China. The biosurfactant was originally received as a blend of 2/3 mono-rhamnolipid and 1/3 di-rhamnolipid, and no further alteration was performed. It is a brown viscous liquid with approximate purify of 40%. The nonionic surfactant TX100 was with purity higher than 98%, and phenanthrene was with purity greater than 97%, both substances were obtained from Tokyo Chemical Industry Co., Ltd., Tokyo, Japan. Acetic acid was purchased from J&K Scientific Ltd., Beijing, China. The selected properties of above substances are shown in Table 1.

### 2.2. Surface Tension Measurements

The CMC values of the individual and blended Triton X-100 and rhamnolipids solution were determined by the DuNuoy ring method [25]. A series of surfactants concentration from 10 to 3000 mg/L were prepared. The mass ratios of TX100 and Rham were enacted at 1:0, 9:1, 3:1, 1:1, 1:3, and 0:1. Surface tension of solutions was measured at 20 °C by the BZY-102 model tensiometer with a 9.55 mm radius platinum ring (Shanghai Fangrui Instrument Co., Ltd., Shanghai, China).

### 2.3. Solubilization Experiments

A stock solution was prepared by dissolving individual Triton X-100 or rhamnolipids in water. The stock solution was diluted to 3000, 2500, 2000, 1500, 1000, 500, and CMC mg/L, respectively. The mass ratios of TX100 and Rham were enacted at 1:0, 9:1, 3:1, 1:1, 1:3, and 0:1.

The solubility of phenanthrene was measured in individual or mixed surfactant solutions. For each batch test, excess amount (crystalline phenanthrene was observed after experiment) phenanthrene was added to glass flasks containing a series of 50 mL individual or mixed surfactant solutions to ensure maximum solubility.

These samples were set for the HZQ-F160 model reciprocating shaker (Changzhou Putian instrument manufacturing Co., Ltd., Changzhou, China) at approximately 200 rpm at 20 °C for 24 h. After shaking, the beakers were centrifuged with LD-5 model centrifuge (Changzhou Putian instrument manufacturing Co., Ltd., Changzhou, China) at 1666 g for 20 min. After solubilization experiments, the samples were taken with 10 mL glass syringes and filtered through a 0.2 μm poly-tetrafluoroethylene filter. Filtered samples were then analyzed with high-performance liquid chromatography (HPLC, Dionex U3000, Sunnyvale, CA, USA). The specific experimental conditions have been described in our earlier study [26]. All the experiments performed in triplicate and the average value is taken as the calculated value. To study the solubilization properties of mixed surfactant in different environment, the effect of pH, ionic strength, and acetic acid were performed. To investigate the effect of pH, the solubility tests were conducted with mixed surfactant concentration at CMC, 500, 1000, 1500, 2000, 2500 and 3000 mg/L, and the mass ratio between TX100 and rhamnolipids was 3:1. The ration value was chosen in consideration of reducing the use of synthetic surfactants and better solubility. The pH value of solution was settled at 5, 6, 7, 8, and 9 by adjusting with buffer solutions. To investigate the effect of ionic strength or organic acids, the solubility tests were conducted in mixed surfactant with different NaCl or organic acids concentrations (5, 10, and 15 mg/L). Then excess amount phenanthrene was added as the batch solubilization experiments described above.

### 2.4. Data Analysis

#### 2.4.1. CMC of Mixed Surfactant Solutions

Based on a simple phase separation model with the assumption of ideal mixing of surfactants in the micelles, the CMC of the mixture (*CMC*_12,theor_) can be predicted as proposed by Clint [27] by the following:

(1) $$\frac{1}{CMC_{12,\mathrm{theor}}}=\frac{\alpha_{1}}{CMC_{1}}+\frac{1-\alpha_{1}}{CMC_{2}}$$

where α_1_ is the bulk mole fraction of TX100 in the binary surfactants, *CMC*_1_ is the critical micelle concentration of TX100, *CMC*_2_ is the critical micelle concentration of rhamnolipids, and *CMC*_theor_ is the critical micelle concentration of ideal mixed surface agent.

#### 2.4.2. Interaction Parameter

Rubingh [28] proposed that the composition of the mixed micelle and the interaction parameter *β*, can be calculated by the following:

(2) $$\beta =\frac{\ln(\alpha_{1}cmc_{12}/X_{1}cmc_{1})}{{\left(1-X_{1}\right)}^{2}}=\frac{\ln[(1-\alpha_{1})cmc_{12}/(1-X_{1})cmc_{2}]}{{\left(X_{1}\right)}^{2}}$$

(3) $$\frac{{\left(X_{1}\right)}^{2}\ln(\alpha_{1}cmc_{12}/X_{1}cmc_{1})}{{\left(1-X_{1}\right)}^{2}\ln[(1-\alpha_{1})cmc_{12}/(1-X_{1})cmc_{2}]}=1$$

where *X*_1_ is the mole fraction of TX100 in the binary surfactants; *CMC*_1_ and *CMC*_2_ is the critical micelle concentrations for TX-100 and rhamnolipids, respectively, and *β* is the surfactants interaction parameter, *CMC*_12_ is the *CMC* experimentally determined for the mixture.

#### 2.4.3. Weight Solubilization Ratio

A useful way to evaluate the effectiveness of a surfactant in solubility enhancement of phenanthrene is to define the weight solubilization ratio (WSR), which be calculated by the following [29]:

(4) $$WSR=\frac{S_{ac}-S_{cmc}}{C_{surf}-CMC}$$

where *S_ac_* is the solubility of phenanthrene at a specified surfactant concentration; *S_cmc_* is the apparent solubility of phenanthrene at *CMC*; *WSR* is the weight solubilization ratio of experimental mixture surfactants, *C_surf_* is the surfactant concentration.

To illustrate the synergetic solubilization ability of surfactant mixture, *WSR*_theor_ can be calculated by the following:

*WSR*_theor_ = *WSR*_1_*m*_1_ + *WSR*_2_*m*_2_ (5)

where *WSR*_theor_ is the *WSR* value for phenanthrene in mixed TX100/Rham mixture system at the ideal binary state. *WSR*_1_ and *WSR*_2_ are the weight solubilization for TX100 and Rham. *m*_1_ and *m*_2_ are the mass fraction of TX100 and Rham in the mixed surfactant system, respectively.

#### 2.4.4. Partition Coefficient *K_m_*

The partition coefficient *K_m_* is defined as the distribution of organic compounds between the micelle and the aqueous phase, which can be calculated by the following formula [30]:

(6) $$K_{m}=\frac{S_{ac}-S_{cmc}}{C_{surf}-CMC+S_{ac}-S_{cmc}}\frac{1}{S_{cmc}*V_{w}}$$

where *K_m_* represents the partition of the organic compound between micelles and the aqueous phase, *V_w_* represents the molar volume of water.

#### 2.4.5. Partition Coefficient *K_m_*_12_

To obtain the partition of phenanthrene between micelle and surfactant mixture, the partition coefficient *K_m_*_12_ is defined as partition of the neutral nonpolar organic solute between micelle and aqueous phase in a mixed surfactants system, which can be calculated by the following [31]:

(7) $$\ln K_{m12}=X_{1}^{m}\ln K_{m1}+X_{2}^{m}\ln K_{m2}+BX_{1}^{m}X_{2}^{m}$$

where *K_m_*_12_ is the micelle–water partition of the solute in mixed surfactants systems, *K_m_*_1_ is the micelle–water partition of the solute in TX100, *K_m_*_2_ is the micelle–water partition of the solute in rhamnolipids, and *B* represents the empirical parameter including inter-surfactant and surfactant-solute interaction.

#### 2.4.6. Standard Free Energy Change ΔGs

The standard free energy change Δ*Gs* (J/mol) during the process of phenanthrene molecules incorporation into micelles can be represented by the following expression [32]:

Δ*G_s_ = −RTlnK_m_* (8)

where *R*, *T* and *K_m_* are the universal gas constant, the absolute temperature, and the micelle-water partition coefficient respectively; Δ*G_s_* represents the standard free energy change.

## 3. Results and Discussion

### 3.1. Interfacial Properties of TX100/Rham Individual and Mixed Solutions

**Figure 1:**
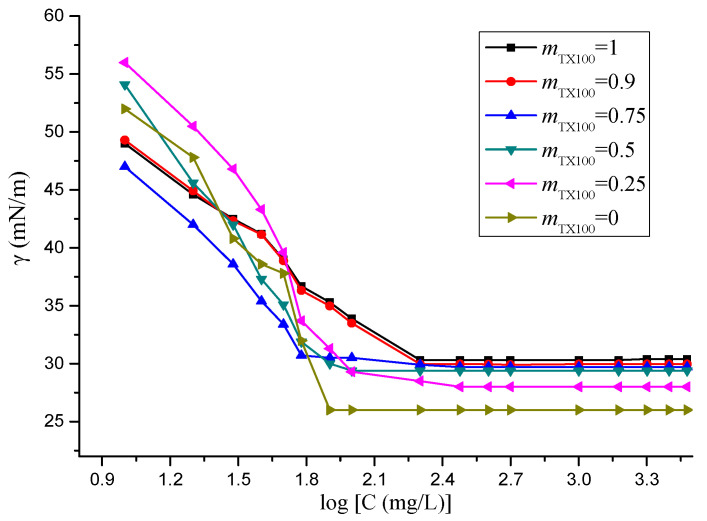
Plots of surface tension as a function of the logarithm values of the total surfactant concentration of the single and TX100–Rham mixture at 10, 20, 30, 40, 50, 60, 80, 100, 200, 300, 400, 500, 1000, 1500, 2000, 2500, 3000 mg/L and with mass ratios of TX100 and Rham at 1:0; 9:1; 3:1; 1:1; 1:3; and 0:1, respectively. (*γ*: surface tension; *C*: total surfactant concentration; *m*_TX100_: the bulk mass fraction of TX100 in TX100 and rhamnolipids (Rham) mixture).

The aqueous surface tension as a function of surfactant concentration for the individual and mixed surfactants is shown in Figure 1. In the left region of the figure, the surfactants exist in a monomer state and tend to accumulate at the interface between air and water. In the right region of the figure, surfactant micelles formed and the surface tension value does not change. At the intersection point of the two lines, surfactant molecules start forming aggregates in the solution to minimize the free energy of the solution. The concentration at beginning of the intersection point of the two lines in the figure is called CMC. The CMC for each surfactant was determined. The mixing behavior between TX100 and rhamnolipids was expected to be nonideal for their different types of headgroups. *CMC*_theor_ can be calculated by Equation (1) and is listed in the Table 2. It is obvious that the *CMC*_exp_ of the mixed surfactants increased with the decrease in the mass fraction of rhamnolipids and were smaller than the *CMC*_theor_ due to the synergistic effect. This negative deviation indicates the existence of interactions between two surfactants in the mixed aggregates. The addition of small amounts of TX100 led to a reduction in the electrostatic self-repulsion between the rhamnolipids head groups [33].

To show the interaction between Rham and TX100, the interaction parameter β was calculated by Equations (2) and (3) (results presented in Table 2). β indicates synergism extent of mixed surfactant micelle, and the value of positive, negative and zero indicates repulsive, attractive and ideal mixed micelle formation, respectively. The β values often is in the range −6~0 for ionic/nonionic mixed surfactants system [18]. The higher value of the interaction parameter, the stronger the mixing nonideality. All β values are negative in the study, which means that the attractive interaction force between the Rham and TX100 after mixing is stronger than the interaction force between two single surfactants. Moreover, the balance between the surfactant molecular interactions depends on both hydrophobic and hydrophilic interactions between the two surfactants. As the two surfactants have similar hydrocarbon chain lengths (C9 for TX100 and C8 for Rham), the negative β value in this system indicates an increase in the electrostatic force between the hydrophilic head group of TX100 and the rhamnolipids, and the surfactant molecules in the micelle form exhibit higher thermodynamic stability than the monomer. The rise in the absolute value of β resulted in more pronounced mixing nonideality. The strongest interaction is occurred at *X*_TX100_ = 0.75 due to the electrostatic forces between the headgroups. *X*_2_ (the mole fraction of rhamnolipids in the mixed surfactants system) was generally higher than *α*_2_, which may be due to the easier formation of micelles by rhamnolipids as evident from its lower CMC value [34].

### 3.2. Solubilization of Phenanthrene by Individual or Mixed Surfactants

**Figure 2:**
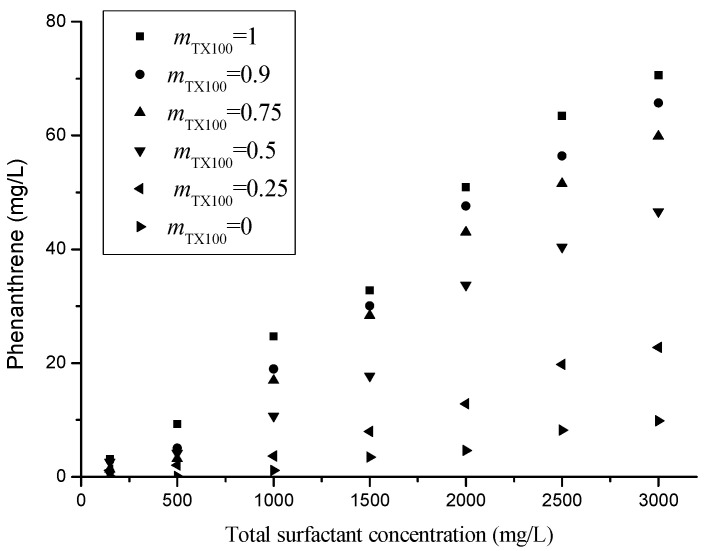
Solubilization of phenanthrene with total surfactant concentration at CMC, 500, 1000, 1500, 2000, 2500 and 3000mg/L, and with mass ratios of TX100 and rhamnolipids at 1:0; 9:1; 3:1; 1:1; 1:3; and 0:1, respectively (*m*_TX100_: the bulk mass fraction of TX100 in TX100 and rhamnolipids mixture).

The solubility of phenanthrene in the presence of a single or mixed surfactant is shown in Figure 2. The solubility of phenanthrene in micelles increases linearly with increasing surfactant concentration. The WSR are calculated by Equations (4) and (5), and are listed in Table 2. The individual TX100 had the highest WSR values, and they decreased with the increase in mass ratios of rhamnolipids in the mixed surfactant systems, which is consistent with the results obtained by Liang et al. [35] in the mixed TX100 and sodium dodecyl sulfate surfactant systems. It should be noted that the WSR value of rhamnolipids in the study is lower than the ones achieved in other publications, which may be attributed to the different qualities and purity of the rhamnolipids used. CMC and *K*_m_ are two key factors affecting the solubilization capability of blended surfactants. *K*_m_ can be calculated by Equation (6) and is listed in Table 2. Log *K*_m_ is greater than Log *K*_ow_ (*K*_ow_ is the partition coefficient for octanol/water), which shows that the apparent solubility of phenanthrene is higher in the micelles than its solubility in the octanol.

The experimental and theory values of *WSR* are also listed in Table 2. All the values of *WSR*_exp_ are bigger than values of *WSR*_theor_ for the same TX100/Rham mixture ratio, which indicates the surfactant mixture have synergetic solubilization ability. The experimental values of apparent solubility are bigger than that of the theoretical values, indicating TX100 and rhamnolipids mixture has synergistic effect. This means that that when rhamnolipids are present, the solubility power of TX100 in the mixture is enhanced due to a synergic effect. In contrast to the ideal mixed effect, there is a deviation between the experimental value and the theoretical value and the solubility data were presented as “nonideality results”. A non-toxic surfactant of rhamnolipids, to partially replace the use of toxic surfactant of TX100, has practical meaning for environmental remediation.

Mixed surfactant systems with more negative β values often have the potential to reduce the solubilization of organics in the micellar phase. Packed micelle and micelle with negative β values can resist the entrance of the phenanthrene molecule from the aqueous phase into the micellar phase [36]. Thus, surfactants with lower CMC and β values should have a better solubilization capability. However, the CMC and β values of the mixed surfactants are not expected to be consistent with WSR values. The mixed surfactants with lower CMC and β values exhibit poor solubilization for phenanthrene as evidenced by its relatively small WSR values obtained in this study, which are consistent with those in the previous literature [35,36,37,38,39]. The results indicate CMC or β is not the parameters for the solubilization of phenanthrene in the surfactant mixture. There is no direct relationship between the solubility and CMC or β. Thus, β values cannot be considered a universal factor responsible for the solubilization behavior. The solubilization of phenanthrene depend on the single surfactant type and structure, solute hydrophobicity, binary surfactant mixture behavior, and surfactant- phenanthrene interaction. As for surfactant mixture, the complicated interaction between the biosurfactants (Rham) and the synthetic surfactant (TX100) in the microenvironment, which influences the number and radius of mixed surfactant micelles, and further affect the solubilization of phenanthrene. Using electron microscopy, the mono-rhamnolipid displayed round or oval shape with relatively uniform size distribution, and di-rhamnolipid showed spherical vesicles and displayed irregular shapes during fluctuations or transitions [40,41]. Mixture of two rhamnolipids showed multilamellar profiles and heterogeneous sizes [40]. Considering organic matter in nature existing in different sizes and shapes, the structure of mixture surfactants may favorable for the solubilization of hydrophobic organic compounds.

Based on thermodynamic ground, the partition coefficient of a neutral organic solute between micellar and aqueous phases (*K*_12_) and B in the mixed surfactant solution were investigated. B values were calculated by Equation (7) and listed in the Table 2. The calculated B values are all positive, suggesting that the mixed surfactant exerted a synergistic solubilization effect on phenanthrene. There is no direct relation between B and β, but both positive B and negative β values have been shown to be associated with the enhanced solubilization capacity of the mixed surfactant. The knowledge of the thermodynamic parameters controlling solubilization helps to better understand the thermodynamics of the processes concerned. The entire solubilization behavior of micelles can be evaluated by the standard free energy. Since R and T are constant, it can be seen from Equation (8) that ΔGs value depends on the *K*_m_ value. All *K*_m_ values were greater than 1 and the ΔGs value must come out to be negative, which shows the spontaneity of the solubilization process.

Generally, mixed surfactant systems have better solubilization and desorption capabilities than single surfactants due to the synergistic effect. There are some similar studies, which are consistent with the results of this study. The surfactant mixtures of tetradecyldimethylammonium bromid and sorbitan monolaurate exhibited better desorption for polycyclic aromatic hydrocarbons contaminated soil [42]. The mixed surfactant of sodium laurate and tween 80 could enhanced water solubilization of polycyclic aromatic hydrocarbons and improve the desorption of naphthalene and phenanthrene from solid matrices [43]. The mixed surfactant of t-octylphenoxypolyethoxyethanol and sodium dodecyl benzene sulfonate could enhance solubilization and desorption of pyrene from soils [39].

The accidental spillage, leakage and inappropriate use of diesel causes soil contamination, and soil usually contains many organic compounds after the relocation of a chemical plant, steel plant or metallurgical plant. These contaminated soils need to be repaired and this study would be significant to those interested in soil remediation method using surfactants for soil washing or flushing.

### 3.3. Effect of pH on Solubilization

**Figure 3:**
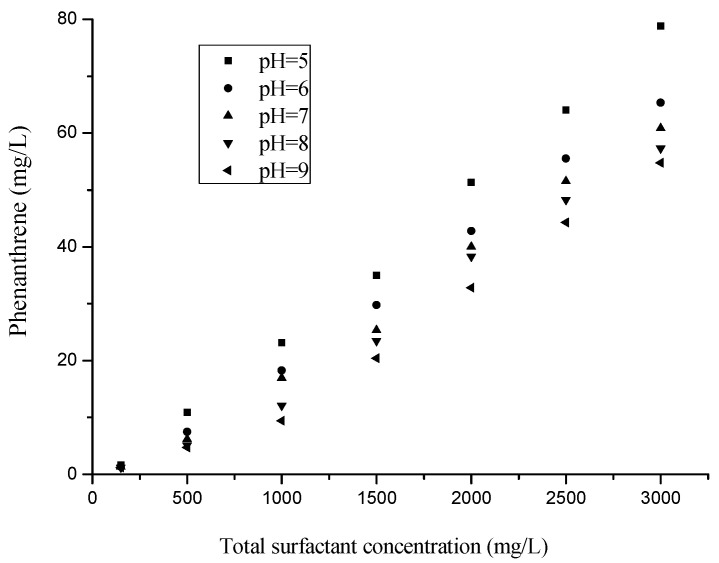
Solubilization of phenanthrene with different pH value (5, 6, 7, 8, 9) in the TX100–Rham mixture at CMC, 500, 1000, 1500, 2000, 2500 and 3000 mg/L (the mass ratio between TX100 and rhamnolipids was 3:1).

The values of pH often have a great influence on the solubilization behavior; thus, the effects of pH on the solubilization capabilities of mixed surfactants at a fixed mass ratio were investigated in the study. The pH values were adjusted by buffer solutions, and the mass ratio between TX100 and rhamnolipids was 3:1. This is because the WSR value relatively high, and stronger interaction between surfactants at *X*_TX100_ = 0.75. The CMC values of the mixed surfactants solutions were 67, 88, 124, 136, and 152 mg/L at pH = 5, 6, 7, 8, and 9, respectively. Therefore, the CMC values displays an obvious trend for an increase with the increase of pH value. However, the WSR and apparent solubilities values in the aqueous phase did not exhibit the same trend with the CMC value. The apparent solubilities of phenanthrene at a total surfactant concentration of 3 g/L were 79.85, 63.35, 60.86, 57.27, and 54.76 mg/L at pH 5, 6, 7, 8, and 9, respectively. The apparent solubilities dropped with the rise of pH of the solution in Figure 3. The WSR values in the mixed surfactant solutions decreased with the rise in the solution pH from 5 to 9.

The value of pH has a great influence on ionic surfactant structures and have little influence on the non-ionic surfactant structures [44]. At a value of the weight ratio TX100: rhamnolipids of 3:1, TX100 is the predominant part in the mixed surfactant. As TX100 is a nonionic surfactant, the pH effect on the a nonionic/anionic micelles structure was mainly determined by the ionic surfactant. Thus, the increase in pH values causes changes to the structure of rhamnolipids, which further influences their solubilization capabilities. Rhamnolipids contain the carboxylic group in their molecules, and the reported *pK*_a_ for rhamnolipids is 5.6. Using transmission electron microscopy, Champion et al. [45] observed a decrease in the size of the structure of rhamnolipids with the increase of pH from 5.5 to 8.0. This is mainly due to the prevalence of the ionized form with a negative charge and the increased repulsion between adjacent polar heads on the micelle surface at pH above 5.6 [46]. Thus, the elevated pH values of the solution would abate the solubilization abilities of phenanthrene in the mixed surfactant. In addition, the negatively charged rhamnolipids have a much higher CMC value, which may cause a considerable decrease in solubility. At pH values lower than 5, rhamnolipids could inhabit their solubilization capabilities due their acid forms. Furthermore, the low pH of the surfactant solution may have a negative impact on the basic property of the soil [47]. The results indicated that the pH of the solution exerted a substantial influence on the solubilization potential of the biosurfactant for phenanthrene. Therefore, the mixed TX100/Rham micelle structures formed at a pH value of around 5.6 are favorable to the solubility of phenanthrene.

### 3.4. Effect of the Ionic Strength on Solubilization

**Figure 4:**
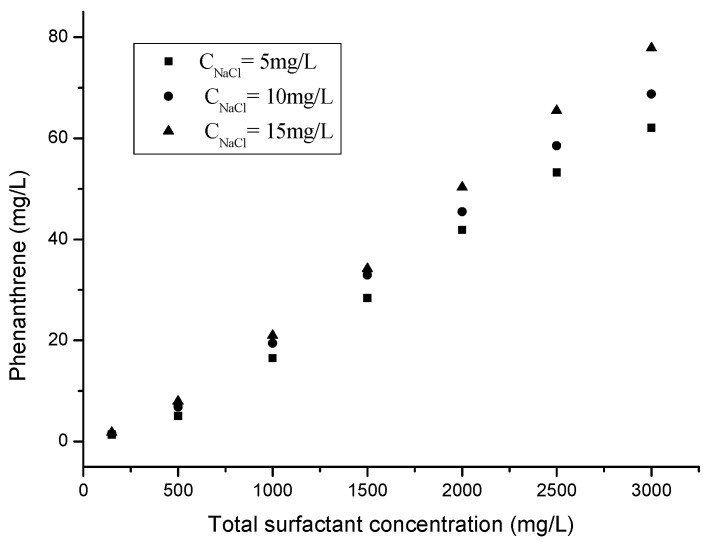
Solubilization of phenanthrene with coexisting NaCl (5, 10, and 15 mg/L) in the TX100–rhamnolipids mixture at CMC, 500, 1000, 1500, 2000, 2500 and 3000 mg/L (the mass ratio between TX100 and rhamnolipids was 3:1, and C_NaCl_ is the concentration of NaCl).

There are many different electrolytes existing in soil and underground water, which may influence the solubilization capabilities of the surfactant utilized. Thus, the effects of ionic strength on the solubilization capabilities of mixed surfactants at a fixed mass ratio were investigated in the study with different NaCl concentrations. The solution salinities were set at 5, 10, and 15 mg/L and the mass ratio of TX100 and rhamnolipids was 3:1. The CMC values of the mixed surfactants solutions were 109, 94, and 81 mg/L at Na^+^ = 5, 10, and 15 mg/L, respectively. The CMC values of the blended surfactant solutions were reduced with the elevation in NaCl concentrations, which resulted in increased solubilization capabilities for phenanthrene of the mixed surfactant solution. The effect of NaCl on phenanthrene solubilization is illustrated in Figure 4, from which it can be observed that the apparent solubilities and WSR of phenanthrene increased with the NaCl concentrations.

There are two possible explanations for this phenomenon. First, with the small addition of Na^+^, the glycolipids hydrophilic group with a negative charge is neutralized, the electric double layer of the micelle is compressed, reducing the mutual repulsion between the ionic heads of the surfactants [48]. The number of aggregated mixed micelles increased, which enhanced the solubilization capabilities. The Log*k_m_* values of the mixed surfactants solutions were 3.09, 3.13, and 3.18 mg/L at Na^+^ = 5, 10, and 15 mg/L, respectively. These results show that the increased ionic strength can arise the apparent solubilities of phenanthrene. Second, the increase in the concentration an electrolyte (NaCl) added to the surfactant solution could lower the CMC value, following the Corrin-Harkins equation [49,50]. The addition of an electrolyte has a salting-in effect, and the solubilization of PAHs enhanced by the non-ionic surfactant. However, it is not possible to increase considerably NaCl concentrations due to at least two disadvantages caused by such a rise. One of them is the possible decrease in the solubility of PAH by the salting-out effect, and the other is the formation of white precipitates in the mixed surfactant system, which hampers the solubilizing capacity. These findings are similar to the study of Song et al. [36]. Adding appropriate amounts of electrolytes to increase the solubilization capabilities of surfactant solutions, and thus greatly reducing the amount of the surfactant, is a common practice in surfactant-enhanced remediation.

### 3.5. Effect of Organic Acids on Solubilization

**Figure 5:**
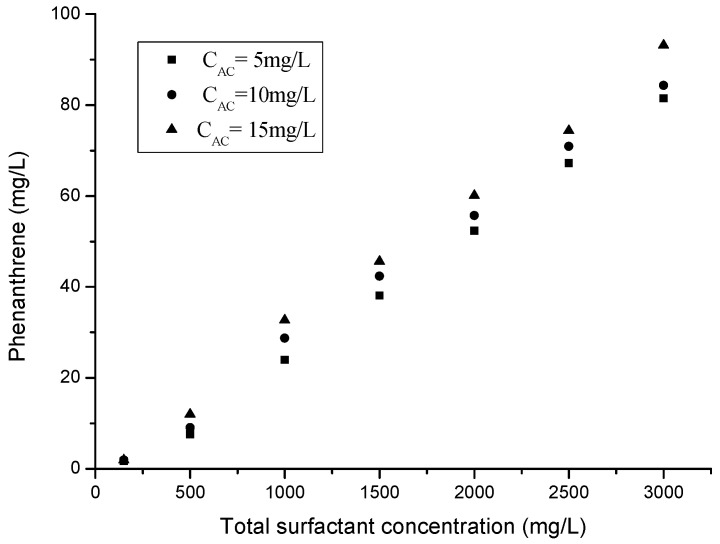
Solubilization of phenanthrene with coexisting acetic acid (5, 10, and 15 mg/L) in the TX100-Rham mixture at CMC, 500, 1000, 1500, 2000, 2500 and 3000 mg/L (the mass ratio between TX100 and Rham was 3:1, and C_AC_ is the concentration of acetic acid).

Organic acids, such as oxalic, succinic, tartaric, and acetic acid occur widely in the soil environment, and they have a considerable effect on the solubilization capabilities of surfactants. Hence, the capabilities of mixed TX100/Rham system for phenanthrene using 5, 10, and 15 mg/L of acetic acid and a mass ratio between TX100 and rhamnolipids set at 3:1 was examined. The CMC values of the mixed surfactants solutions were 105, 85, and 72 mg/L at 5, 10, and 15 mg/L of acetic acid used, respectively. The CMC values of the mixed surfactant solutions were reduced with the increase in acetic acid concentrations, which could enhance the apparent solubilities of phenanthrene. The effect of solution acetic acid on phenanthrene solubilization is illustrated in Figure 5, from which it can be observed that the apparent solubilities and WSR of phenanthrene rose with the elevation of acetic acid concentrations.

Some possible explanations for this phenomenon are the following. First, acetic acid tended to diffuse and dissociate into the water, resulting in a decrease in the pH of the solution. Acetic acid was more susceptible to dissociation due to its higher polarity, which lowered the CMC values of the mixed surfactant and subsequently increased the solubilization capabilities. Second, acetic acid could be distributed into the surfactant tails, which would influence the mixed surfactant micelles structure. This high amount of organic acid close to the interfacial film makes it softer and more flexible, promoting the formation of a more complex structure and helping to solubilize the hydrophobic organic compounds [51]. Therefore, organic acids act as a co-surfactant and enhance the solubilization capabilities. Moreover, organic acids can perform more important functions in real practice. Some studies revealed that organic acids can significantly promote the release of PAHs and improve the availability of PAHs in soil [52]. Thus, adding a suitable amount of an organic acid is beneficial to the process of surfactant-enhanced remediation.

## 4. Conclusions

The phenanthrene solubilization in a TX100/Rham mixture system was evaluated. Surface tension and micellar properties were evaluated by theoretical models. Mixed surfactant solutions show nonideality results as indicated by CMC and β values. The obtained β values and B values indicate that the solubilizing power of all mixed surfactant systems is improved. Solubilization capabilities increased with decreasing mass ratio of rhamnolipids in mixed micelles. The solubilization capabilities of the TX100/Rham mixture system for phenanthrene declined with the increase in the pH values of the solution from 5 to 9, but rose with the elevation of ionic strength and acetic acid concentrations. The mixtures of rhamnolipids and synthetic surfactants show a synergistic behavior and enhanced solubilization capabilities. Thus, the application of mixtures of rhamnolipids and synthetic surfactants will extend the use of these environmentally friendly biosurfactants and enhance their accessibility and cost-effectiveness, which will broaden the prospects for their application in the remediation of organically contaminated soil.

## Figures and Tables

**Table 1:** Selected properties of substances in this study.

	Molecular Formula	Molecular Structure	Molecular Weight (g/mol)	Water Solubility 25 °C	Critical Micelle Concentration mg/L
phenanthrene	C_14_H_10_	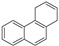	178	1.2 mg/L	
mono-rhamnolipid	C_26_H_48_O_9_	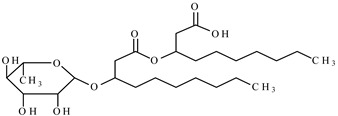	504		50
di-rhamnolipid	C_32_H_58_O_13_	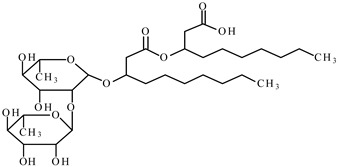	650		98
Triton X-100	C_8_H_17_C_6_H_4_O(OCH_2_CH_2_)_9.5_H	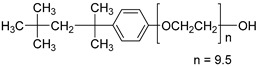	625		150
acetic acid	CH_3_COOH	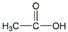	60		

**Table 2:** Solubilization parameters, critical micelle concentration CMC, interaction parameter, weight solubilization ratio (WSR), micelle-water partition coefficient (Km), and free energy of solubilization of phenanthrene in the mixed TX100-Rham system with different mass ratios of TX100 and rhamnolipids at 1:0; 9:1; 3:1; 1:1; 1:3; and 0:1, respectively.

*m*_TX100_	*α*_1_	*CMC*_theor_	*CMC*_exp_	*X*_1_	*β*	*WSR*_theor_	*WSR*_exp_	ln*K*_12_	*B*	*G*
1	1		0.309				0.025	6.892		−17.02
0.9	0.888	0.270	0.253	0.744	−0.379	0.022	0.024	7.036	0.225	−17.37
0.75	0.726	0.228	0.204	0.528	−0.432	0.019	0.022	7.089	0.156	−17.51
0.5	0.469	0.182	0.175	0.294	−0.206	0.014	0.017	7.197	0.283	−17.77
0.25	0.227	0.154	0.149	0.136	−0.291	0.007	0.008	7.232	0.352	−17.86
0	0		0.134				0.004	7.237		−17.87

## Nomenclature

m_TX100_	the bulk mass fraction of TX100 in the binary surfactants
α_1_	the bulk mole fraction of TX100 in the binary surfactants
β	surfactants interaction parameter
γ	surface tension (N/m)
CMC	critical micelle concentration (mol/L)
CMC_1_	the CMC value of TX100 (mol/L)
CMC_2_	the CMC value of rhamnolipids (mol/L)
*CMC*_theor_	the CMC value of ideal mixture surfactants (mol/L)
*CMC*_exp_	the CMC value of experimental mixture surfactants (mol/L)
$X_{1}$	the mole fraction of TX100 in the binary surfactants
$X_{2}$	the mole fraction of rhamnolipids in the binary surfactants
*WSR*_exp_	the weight solubilization ratio of experimental mixture surfactants
*WSR*_theor_	the weight solubilization ratio of deal mixture surfactants
*S_ac_*	the solubility of phenanthrene at a specified surfactant concentration (mg/L)
*S_cmc_*	the apparent solubility of phenanthrene at *CMC* (mg/L)
*C_surf_*	surfactant concentration (mg/L)
*V_w_*	the molar volume of water (L/mol)
*K_m_*	the partition of the organic compound between micelles and the aqueous phase
K_m12_	the micelle–water partition of the solute in mixed surfactants systems
K_m1_	the micelle–water partition of the solute in TX100
K_m2_	the micelle–water partition of the solute in rhamnolipids
ΔG_s_	the standard free energy change (J/mol)
R	the universal constant of the gas (J/(mol·K))
T	temperature (K)
B	an empirical parameter including inter-surfactant and surfactant-solute interaction
*pK_a_*	acidity coefficient
